# Influence of gastrocnemius-soleus muscle force on sub-MTH load distribution

**DOI:** 10.1186/1757-1146-5-S1-O26

**Published:** 2012-04-10

**Authors:** Wen-Ming Chen, Victor Phyau-Wui Shim, Taeyong Lee

**Affiliations:** 1 Division of Bioengineering, National University of Singapore, Singapore; 2 Department of Mechanical Engineering, National University of Singapore, Singapor

## Background

The gastrocnemius-soleus (G-S) muscle complex, the most dominant extrinsic plantar flexor, plays an important role in the normal weight-bearing function of the foot. The stability and stance-phase placement of the foot can be adversely affected when muscular loads/support are abnormal (e.g. equinus contracture) [1]. This study aims to formulate a three-dimensional musculoskeletal finite element (FE) model of the foot to quantify the influence of G-S muscle force on forefoot metatarsal head (MTH) load distribution.

## Materials and methods

The FE model established corresponds to a muscle-demanding posture in heel-rise, with simulated activation of major extrinsic plantar flexors. In a baseline case, the required muscle forces were inversely determined from what would be necessary to generate the targeted ground reaction forces corresponding to known boundary conditions. This baseline model served as a reference for subsequent parametric analysis. The adaptive changes of the foot mechanism (i.e., internal joint configurations and plantar loads distributions) in response to decreased muscle forces in G-S complex (adjusted in a step-wise manner) were analyzed. The muscle force adjustments mimic effects of surgical tendo-Achilles lengthening procedure [2].

## Results

**Figure 1:**
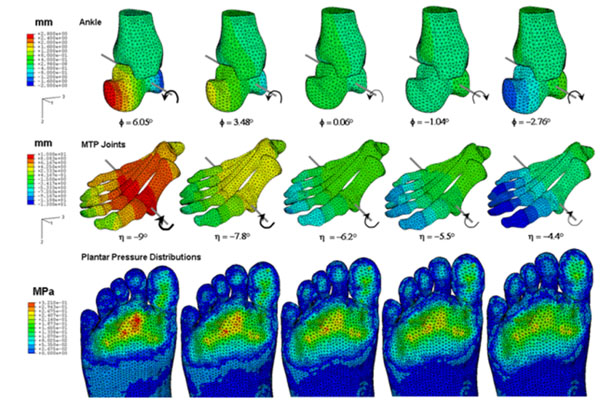
Changes in plantar pressure distribution for different angles at ankle and MTP joints in response to reduction of the gastrocnemius-soleus muscle force.

Movements of the ankle and metatarsophlageal joints, as well as the forefoot plantar pressure peaks and the pressure distribution under the metatarsal heads were all found to be extremely sensitive to reduction in the muscle load in the G-S complex. A 40% reduction in G-S muscle stabilization can result in dorsal-directed rotations by 8.81° at the ankle and decreased metatarsophalangeal joint extension by 4.65° (Figure 1). The resulting peak pressure reductions at individual MTHs, however, may be site-specific and possibly dependent on foot structure, such as intrinsic alignment of the metatarsals.

## Conclusions

The relationships linking muscular control, internal joint movements, and plantar loading distributions are envisaged to have important clinical implications on tendo-Achilles lengthening procedures, and to provide surgeons with an understanding of the underlying mechanism for relieving forefoot pressure in diabetic patients suffering from ankle equinus contracture.
